# Discovery of anti-infective compounds against *Mycobacterium marinum* after biotransformation of simple natural stilbenes by a fungal secretome

**DOI:** 10.3389/fmicb.2024.1439814

**Published:** 2024-09-17

**Authors:** Jahn Nitschke, Robin Huber, Stefania Vossio, Dimitri Moreau, Laurence Marcourt, Katia Gindro, Emerson F. Queiroz, Thierry Soldati, Nabil Hanna

**Affiliations:** ^1^Department of Biochemistry, Faculty of Science, University of Geneva, Geneva, Switzerland; ^2^School of Pharmaceutical Sciences, University of Geneva, CMU, Geneva, Switzerland; ^3^Institute of Pharmaceutical Sciences of Western Switzerland, University of Geneva, CMU, Geneva, Switzerland; ^4^ACCESS Screening Platform, NCCR Chemical Biology, Faculty of Science, University of Geneva, Geneva, Switzerland; ^5^Mycology Group, Research Department Plant Protection, Agroscope, Nyon, Switzerland

**Keywords:** anti-infectives, natural products, phenotypic screening, *Mycobacterium marinum*, *Dictyostelium discoideum*, stilbene derivatives

## Abstract

**Introduction:**

*Mycobacterium tuberculosis* (Mtb), the causative agent of tuberculosis, remains a serious threat to human health worldwide and the quest for new anti-tubercular drugs is an enduring and demanding journey. Natural products (NPs) have played a significant role in advancing drug therapy of infectious diseases.

**Methods:**

This study evaluated the suitability of a high-throughput infection system composed of the host amoeba *Dictyostelium discoideum* (Dd) and *Mycobacterium marinum* (Mm), a close relative of Mtb, to identify anti-infective compounds. Growth of Dd and intracellular Mm were quantified by using luminescence and fluorescence readouts in phenotypic assays. The system was first benchmarked with a set of therapeutic anti-Mtb antibiotics and then used to screen a library of biotransformed stilbenes.

**Results:**

The study confirmed both efficacy of established antibiotics such as rifampicin and bedaquiline, with activities below defined anti-mycobacterium susceptibility breakpoints, and the lack of activity of pyrazinamide against Mm. The screening revealed the promising anti-infective activities of *trans*-δ-viniferins and in particular of two compounds **17** and **19** with an IC_50_ of 18.1 μM, 9 μM, respectively. Both compounds had no activity on Mm in broth. Subsequent exploration via halogenation and structure-activity relationship studies led to the identification of derivatives with improved selectivity and potency. The modes of action of the anti-infective compounds may involve inhibition of mycobacterial virulence factors or boosting of host defense.

**Discussion:**

The study highlights the potential of biotransformation and NP-inspired derivatization approaches for drug discovery and underscores the utility of the Dd-Mm infection system in identifying novel anti-infective compounds.

## Introduction

Tuberculosis, an infectious disease caused by *Mycobacterium tuberculosis* (Mtb), has tragically claimed the lives of nearly 30 million people across the globe over the past decade, as reported by the World Health Organization (WHO) (Bagcchi, 2023). The tuberculous mycobacteria within the Mtb complex pose a difficult challenge for drug targeting, as these intracellular pathogens reside inside a vacuole in macrophages. Adding to this complexity, the intricate and waxy mycobacteria cell wall serves as an impermeable barrier to effective antibiotic treatment (Alderwick et al., 2015). The quest for new anti-tubercular drugs is an enduring and demanding journey further complicated by Mtb’s ability to enter a metabolically dormant state. Indeed, latent infections are often accompanied by non-heritable antibiotic tolerance, hindering the efficacy of conventional antibiotics and presenting a complicated challenge in disease control (Lewis, 2007). Adding to the gravity of the situation is the swift emergence of Multi-Drug-Resistant (MDR) and Extensively-Drug-Resistant (XDR) strains, which demand prolonged antibiotic treatment associated with severe side effects, high costs, and a limited chance of cure (Dheda and Migliori, 2012). Regrettably, despite the escalating threat of antibiotic resistance, the implementation of new antibiotics had been declining. However, thanks to a world-wide effort centered in universities, much progress has recently been achieved with the identification of a substantial number of drug candidates and newly approved drugs with novel mechanisms of action (Koul et al., 2007; Makarov et al., 2009; Lu et al., 2019; Alsayed and Gunosewoyo, 2023). Consequently, the development of new regimens for the treatment of drug-susceptible, MDR, and XDR Mtb infections are now conceivable. In fact, in 2019, the first 6-month regimen for the treatment of multidrug-resistant (MDR) and extensively drug-resistant (XDR) TB was approved, consisting of just three drugs with two novel mechanisms of action: bedaquiline, pretomanid, and linezolid (Koul et al., 2008; Conradie et al., 2020). Despite this success, a continuation of the effort to “fill the pipeline” is necessary.

In the pursuit of new anti-Mtb compounds, two primary approaches have been embraced: targeted drug discovery and whole-cell screening. While the main drawback of targeted drug discovery is that the selected target may not be essential *in vivo*, phenotypic screenings have exhibited more promise, giving rise to compounds such as SQ109 which is in clinical trial phase II (Reddy et al., 2010), bedaquiline (Andries et al., 2005), or delamanid (Matsumoto et al., 2006). Phenotypic screens in whole-cell assays offer numerous advantages over target-based drug discovery, including the identification of novel targets, probing multiple targets simultaneously or detection and exclusion of compounds with low membrane permeability. The fact that 40 years passed before bedaquiline was discovered as a new first-in-class drug (Mahajan, 2013), emphasizes the need for new targets and the importance of phenotypic assays.

Even though SQ109, bedaquiline and delamanid were discovered by phenotypic whole-cell screens on mycobacteria in broth, *in vitro* screening neglects that Mtb is an intracellular, vacuolar or cytosolic pathogen. Consequently, screening approaches should better take into account mycobacteria’s infection biology by making use of dedicated host-pathogen systems. Recent efforts shifted their focus towards a variety of *in cellulo* infection models, encompassing both cells of animal and non-animal origin. The experimentally versatile Amoebae – *Mycobacterium marinum* (Mm) infection models provide robust and ethically compliant platforms for investigating mycobacterial pathogenicity (reviewed in Cardenal-Munoz et al., 2018; Dunn et al., 2018; Guallar-Garrido and Soldati, 2024). Crucially, both Mtb and Mm employ highly similar strategies to establish an infection by hindering phagosome maturation and avoiding killing in macrophages and amoebae, while Mm offers experimental advantages, such as faster growth and being an organism requiring biosafety level 2 infrastructure (Ramakrishnan, 2004; Tobin and Ramakrishnan, 2008). On the host side, two main amoebae systems, *Acanthamoeba castellanii* (Kicka et al., 2014; Diop et al., 2018, 2019) and *Dictyostelium discoideum* (Dd), are commonly used to study the infectious process caused by various pathogenic bacteria, including Salmonella, Mycobacteria, Legionella, or Pseudomonas (Solomon et al., 2003; Steinert and Heuner, 2005; Hagedorn and Soldati, 2007; Dunn et al., 2018). Both amoebae are professional phagocytes employing evolutionary conserved strategies to capture and kill bacteria. Similarly to an Mtb infection, after uptake of Mm in macrophages or amoebae, the phagosome becomes a Mycobacteria-Containing Vacuole (MCV), an active interface between the host cell and the mycobacteria, being at the crossroads of host machineries such as membrane damage repair, autophagy, oxidative burst and lysosomal fusion, and virulence factors of the pathogen, such as type VII secretion systems and their effectors. Eventually, the MCV loses its integrity, allowing Mm to access the host cytosol (Cardenal-Munoz et al., 2017; Dunn et al., 2018; Lopez-Jimenez et al., 2018). Amoebae systems and especially Dd offer experimental advantages over animal derived cellular models mainly due to low maintenance cost and ease of genetic manipulation, rendering them ideal platforms for high-throughput approaches. The Dd-Mm system is a powerful 3R model system. The 3R principles were coined in the 1950s and stand for Replacement, Reduction and Refinement of animal experiments (Russell and Burch, 1959).

Direct screening inside an infected host allows to rapidly exclude compounds that are toxic to the host or have poor pharmacokinetics, thereby significantly contributing to decrease the usually high attrition rate in drug screens (Huszár et al., 2020). Most importantly, such systems open the possibility to identify host-targeting compounds that boost cell-autonomous defence and bacteria killing. This strategy termed adjunctive or host-directed therapy (HDT) is of particular interest as a strategy to circumvent resistant strains such as MDR-Mtb (Gries, 2024). For example, bedaquiline has been shown to activate macrophages in addition to its antibiotic effect (Giraud-Gatineau et al., 2020). The drug’s concentration in lipid droplets of the host cell (Greenwood et al., 2019) might play a substantial role in its mode of action and emphasizes the need of considering host biology when searching for new drugs. Besides unlocking host targets, host-pathogen screening systems also have the potential to guide the way to targets within bacterial effectors, which are specific for the intracellular life of mycobacteria (Gries et al., 2020). Compounds with such an activity have been identified by investigating hits from a host-pathogen screen, which were inactive in a screen on mycobacteria (Rybniker et al., 2015).

Even with the amoeba-Mm infection model as a valuable screening tool, the choice of chemical space to screen is crucial to success. In this regard, natural products (NPs) and their derivatives have played a significant role throughout history in advancing drug therapy, with notable contributions in fields such as cancer and infectious diseases (Newman and Cragg, 2016, 2020). The hegemony of NPs in drug discovery was severely challenged in the 1990s, with a major shift towards synthetic and combinatorial chemistry. However, this shift did not deliver the expected success (David et al., 2015). Recent advances in “omics” techniques, computational methods, and innovative screening models have revitalized the field of phenotypic screens in complex systems, and opened up new avenues of exploration (Rossiter et al., 2017; Wolfender et al., 2019). Despite this advance, several challenges in NP drug discovery remain, such as access to novel biological material, intraspecies variability and supply (Kingston, 2011; David, 2018). An alternative approach that takes advantage of the aforementioned advance is the generation of new NP-like compounds based on NP scaffolds. Such a strategy can take advantage of the use of enzymes to generate derivatives of known NPs that are not or not yet found in nature. Biotransformation has a number of advantages over classical organic chemistry. Reactions can take place under mild conditions, near neutral *pH*, ambient temperatures and atmospheric pressure, and protection of certain functional groups is often not required.

Based on this strategy, we recently presented the use of an enriched enzyme fraction secreted by the fungus *Botrytis cinerea* (“enzymatic secretome”) to transform NPs, such as stilbenes, into more complex structures with improved biological activities against fungi, breast cancer, bacteria and viruses (Gindro et al., 2017; Righi et al., 2020; Huber et al., 2022a; Zwygart et al., 2023). This approach yielded a small library of more than 70 compounds distributed in six scaffolds. Since stilbene derivatives have been reported to have potential anti-TB activity in various *in vitro* assays (Suarez et al., 2017; Reinheimer et al., 2018; Peraman et al., 2021), this library was subjected to an anti-infective screening workflow in the Dd-Mm infection model. The goal of this proof-of-concept study was to develop and benchmark the Dd-Mm system to high-throughput capacity in order to screen a focused library of natural products derivatives for anti-infective activities, which theoretically include both antivirulence and host defence-boosting activities that will be disentangled in future studies.

## Materials and methods

### Generation of a stilbene dimer library by chemoenzymatic synthesis

The initial screening library was generated by chemoenzymatic synthesis for a previous study on Wnt pathway inhibition (Huber et al., 2022a). Briefly, compounds **1–4** (99% purity) were purchased from Biopurify Phytochemicals Ltd. (Chengdu, Sichuan, China), and dimers **5–54** were obtained by enzymatic radical dimerization of 1 and 2 using the enzymatic secretome of *Botrytis cinerea* Pers (Gindro et al., 2017). Each compound was isolated by semi-preparative high-resolution liquid chromatography (semi-preparative-HPLC) and characterized by nuclear magnetic resonance (NMR), high-resolution mass spectrometry (HRMS) and ultraviolet (UV) spectroscopy. Details on the synthesis, isolation and characterization of the compounds can be found in their original reference (Huber et al., 2022a).

### Isolation of *trans*-δ-viniferin enantiomers

Both enantiomers of the *trans*-δ-viniferin derivatives **11**, **17**, **19**, and **21** were previously isolated for their antibacterial evaluation against *Staphylococcus aureus* (Huber et al., 2023). This was achieved by using a chiral stationary phase in HPLC. Each enantiomer was characterized by electronic circular dichroism (ECD) and optical rotation to assess its absolute configuration. Details of the isolation and characterization of these enantiomers can be found in the original reference (Huber et al., 2022b; Huber, 2023).

### Generation of *trans*-δ-viniferin derivatives

The collection of *trans*-δ-viniferin derivatives **55–94** was generated for a previous study on antibacterial activity against *S. aureus* (Huber et al., 2023). Several strategies were employed, including (1) light-induced double-bond isomerization, (2) *O*-methylation of the phenolic moieties, (3) oxidation of the dihydrobenzofuran ring to benzofuran, (4) radical dimerization of other stilbene monomers and (5) oxidative halogenation. Each compound was isolated by semi-preparative HPLC and characterized by NMR, HRMS and UV spectroscopy. Details of the synthesis, isolation and characterization of the compounds can be found in their original reference (Huber, 2023).

### Antibacterial assay

Mm WT expressing the *lux* operon (*luxCDABE*) under the *hsp60* promoter (Andreu et al., 2010; Arafah et al., 2013) was cultured for 24 h prior to the experiment at 32°C under shaking conditions in 10 mL 7H9 broth (Becton Dickinson, Difco Middlebrook 7H9) containing 0.2% glycerol (Sigma Aldrich), 10% OADC (Becton Dickinson) and 0.05% tyloxapol (Sigma Aldrich). The Mm culture was centrifuged at 300 RPM for 1 min to sediment large bacterial clumps. The OD_600_ of the supernatant was then measured to determine and subsequently adjust the bacterial density to 3.75*10^5^ bacteria per ml in 7H9 medium. Each well of a 384-well plate (Interchim FP-BA8240) was filled with 20 μL of the bacterial suspension (7.5*10^3^ bacteria), and 0.2 μL of the dissolved compounds were added in a 1:100 dilution to each well to a final vehicle concentration of 0.5% ethanol. Each compound was tested in technical duplicates, and at least 24 technical replicates of control conditions were included: a vehicle control (VC) with a final concentration of 0.5% ethanol and rifabutin at a final concentration of 10 μM as a positive control. The plates were sealed with a gas-impermeable membrane (H769.1, Carl Roth), briefly centrifuged and bacterial growth was monitored using an Agilent BioTek H1 plate reader, an Agilent BioTek BioStack plate stacker, and luminescence was recorded over 72 h at 32°C with readings taken every hour. For every assay plate the robust Z’-factor was calculated using the vehicle control and the positive control. Each plate included 4 positive controls (Rifabutin) and 12 vehicle control wells (0.5% EtOH). The average Z’-factor was calulated as a mean of 3 biological replicates.

### Anti-infective assay

Dd Ax2(ka) expressing mCherry at the *act5* locus (Paschke et al., 2019) was cultured in 10 cm culture dishes (Falcon) in HL5-C medium (Formedium). Mm M strain expressing the bacterial *lux* operon (*luxCDABE*) was cultured at 32°C under shaking conditions in 7H9 broth (Becton Dickinson, Difco Middlebrook 7H9) containing 0.2% glycerol (Sigma Aldrich), 10% OADC (Becton Dickinson) and 0.05% tyloxapol (Sigma Aldrich). The day before the experiment 10^7^ amoebae were plated in a 10 cm Petri dish. On the day of the experiment, infection was performed at MOI 25 by spinoculation in HL5-C and subsequently washing off extracellular bacteria (Mottet et al., 2021). Then, the cell suspension of infected amoebae was detached from the culture dish and resuspended in HL5-C with 5 U/mL penicillin and 5 μg/mL of streptomycin (Gibco) to inhibit extracellular growth of bacteria. The density of infected cells was quantified using a Countess (Thermo Fisher Scientific), and 1 × 10^4^ cells in 20 μL of HL5-C with penicillin and streptomycin were seeded into each well of a 384-well plate (Interchim FP-BA8240). Test compounds resuspended in 50% ethanol were tested in technical triplicates and added in a 1:100 dilution to a final vehicle control concentration of 0.5% ethanol. The plates were sealed (H769.1, Carl Roth), briefly centrifuged and fluorescence and luminescence were monitored using an Agilent BioTek H1 plate reader and an Agilent BioTek BioStack plate stacker in a temperature-controlled environment (set to 24°C) over 72 h with readings taken every hour.

For both assays, growth curves were obtained by measuring the luminescence and fluorescence as a proxy for bacterial growth and host growth, respectively, for 72 h with time-points taken every hour. The “normalized residual growth” was computed by calculating the area under the curve (AUC, trapezoid method) and normalization to the vehicle control (0.5% ethanol, set at 1) and a baseline curve (set at 0). The baseline curve was calculated by taking the median of the first measurement of all wells in a plate and extrapolating it over the full time course. The threshold for hit detection was arbitrarily fixed at a cut-off of normalized residual growth <0.5. Normalized values were averaged over technical and biological replicates (all experiments at least *n* = 3 and *N* = 3) before estimating the IC_50_. For IC_50_ estimation, a robust 4PL regression was used, constraining the top value to 1 and, if necessary, the bottom value to –2. This was performed using GraphPad Prism (Version 8.0.1). The MIC was determined as the lowest experimental concentration, where the averaged normalized residual growth plus one standard deviation was ≤0. For every assay plate the robust Z’-factor was calculated using the vehicle control and the positive control. Each plate included 24 positive controls (Rifabutin) and 60 vehicle control wells (0.5% EtOH). The average Z’-factor was calulated as a mean of 3 biological replicates.

### Readouts of the assays and terminology

To classify the readouts of the antibacterial and infection assays, we coined terms that encompass all the states/populations identified. The growth of Mm in broth is simply defined as “Mm-broth.” The situation is slightly more complex for the infection assay. Our standard protocol leads to the robust and reproducible infection of about 50% of the amoebae with approximately 1–5 bacteria per cell at time zero (Trofimov et al., 2018; Mottet et al., 2021; Bagcchi, 2023). Therefore, “Dd-inf” refers to the monitoring of the mixed infected and un-infected Dd population, which evolves through the infection course. The situation is similar for the Mm populations. At time zero, the number of extracellular Mm is negligeable, but during infection the plate reader assay does not distinguish the intracellular from the extracellular Mm signal (the latter resulting from multiple release mechanisms: exocytosis, host-cell lysis and ejection). Therefore,” Mm-inf” refers to the monitoring of the intracellular and extracellular mixed Mm populations during infection.

### Anti-infective assay with high-content microscopy

The procedure described here was optimized from Mottet et al. (2021). To monitor both the host and pathogen during the course of infection in a quantitative manner, Dd Ax2(ka) cells expressing mCherry knocked-in at the *act5* locus (Paschke et al., 2019) were infected as described above with Mm WT stably expressing GFP (Msp:12-GFP). As described above, the day before the experiment, 10^7^ amoebae were plated in a 10 cm Petri dish. On the day of the experiment, infection was performed, as described above, in HL5-C at MOI 25 by spinoculation. This was followed by eight washes in HL5-C to clear a maximum of non-internalized bacteria. The infected amoebae were detached mechanically and resuspended in HL5-C with 0.75 U/mL penicillin and 0.75 μg/mL of streptomycin (Gibco). Compounds were diluted in HL5-C to a 10x stock of the final assay concentration and pre-plated in 20 μL into the respective wells of a 96-well μ-plate (iBIDI 96-well μ-plate, 89,626). Subsequently, 7,500 cells per well were added in a volume of 180 μL. Every condition has been plated in at least three technical replicates and at least three biological replicates. The 96-well plate was sealed with a gas permeable seal (4titude, 4ti-0516/96) and centrifuged briefly.

Microscopy images were acquired at 25°C with a 40X plan Apo objective at selected time points over 48 h on the ImageXpress Micro C® automated microscope (Molecular Devices) in wide field mode. Focus was obtained automatically by autofocusing on the well bottom and plate bottom, at every well and every time point. In a grid of 4 × 5, 20 images per well, corresponding to a range of 500–6,000 cells, have been taken using the Texas-Red channel for Dd expressing mCherry and FITC channel for Mm expressing GFP in each experiment. Image analysis was performed using MetaXpress Custom Module editor software. Objects were effectively segmented by size and intensity. More precisely, amoebae were segmented using a “find blob module” using the Texas-Red channel. To segment the bacteria, the FITC image was first subjected to a light deconvolution with a top hat filter and then segmented using a “find blob module.” Intracellular bacteria and infected amoebae were determined by associating the mask of the amoebae and the bacteria. Several parameters such as intracellular and extracellular bacterial area sum, amoebae area sum, averaged and integrated fluorescence intensity and feature counts of bacteria and amoeba were extracted. The growth curves for the area sum of uninfected amoebae, infected amoebae and intracellular bacteria of independent biological replicates were averaged and plotted using GraphPad Prism (Version 8.0.1).

## Results

### Benchmarking the Dd-Mm high-throughput system

**Figure 1:**
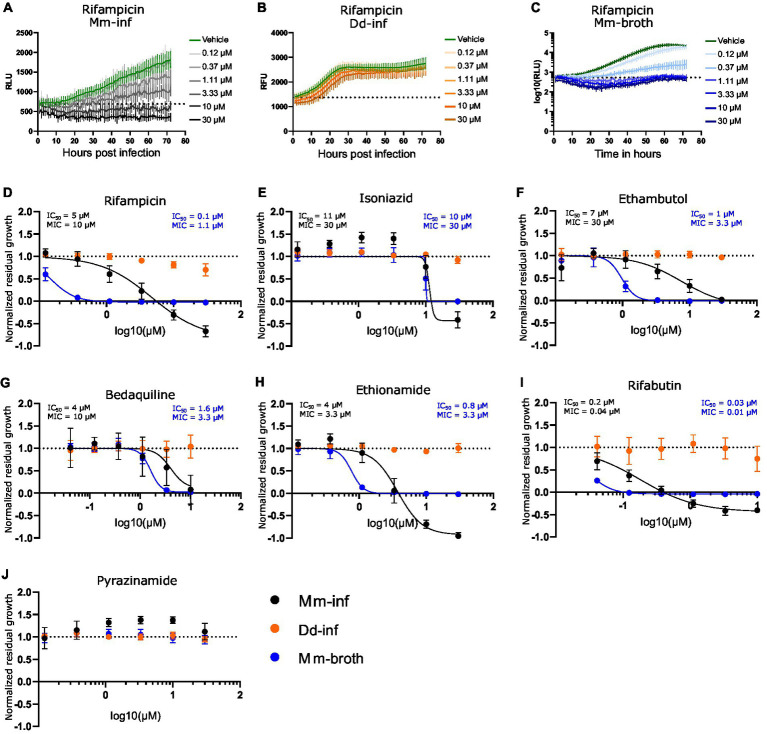
Dose response curves of benchmarked antibiotics A selection of antibiotics used for treatment of a TB infection were benchmarked in the Dd-Mm high-throughput system, in infection and in broth. In panels **(A–C)**, 72 h growth curves of Mm in infection (Mm-inf), Dd in infection (Dd-inf) and Mm in broth (Mm-broth), respectively, at different concentrations of rifampicin are shown. The dashed line represents the median over the first measurements of all wells of the respective experiment. On the *y*-axis are random luminescence units (RLU) or random fluorescence units (RFU). The former were log10 transformed in **(C)**. The corresponding dose–response curve of Rifampicin is shown in **(D)**. The dose–response curves depicted in panel **(E)** isoniazid, **(F)** (ethambutol), **(G)** (bedaquiline), **(H)** (ethionamide), **(I)** (rifabutin) and **(J)** (pyrazinamide) were created from growth curves in analogous manner. Black points show Mm-inf, orange points Dd-inf and blue points Mm-broth. Log_10_ of the test concentration in μM is shown on the *x*-axis. A dashed line at *y* = 1 represents the normalized residual growth of the vehicle control. Depicted are means of at least three biological replicates and the respective standard deviations. Black and blue text inlays show the MIC and IC_50_ of the anti-infective or the antibacterial assay, respectively.

The high-throughput *Dictyostelium discoideum - Mycobacterium marinum* (Dd-Mm) infection model was benchmarked by monitoring dose–response curves (DRCs) for seven antibiotics used in TB chemotherapy (Figure 1). First, we used an anti-infective assay to measure the intracellular growth of a bioluminescent Mm strain (Mm-inf) inside Dd cells expressing mCherry, allowing to also measure in parallel the growth of the host amoebae (Dd-inf). Second, we used an antibiotic assay measuring the growth of Mm in broth (Mm-broth) (see definitions in Materials and methods). In both assays, the luminescence serves as a proxy for Mm mass and metabolic activity. The bacteria or the infected amoebae population were plated in 384-well plates, treated with descending concentrations of each antibiotic and monitored for 72 h using a plate reader. The average robust Z’-factor of the respective benchmarking experiments was 0.73 for Mm-inf and 0.61 for Mm-broth. The antibiotics rifampicin, isoniazid, ethambutol, bedaquiline, ethionamide and rifabutin exhibited sterilizing activity in both assays at their respective maximal tested concentration (Figures 1A–I). In addition, we observed a slight pro-infective effect of isoniazid and pyrazinamide between 0.4 and 3.3 μM (Figures 1E,J), but the latter showed otherwise no activity against Mm (Figure 1J). Moreover, rifampicin and ethambutol showed dramatically different activities in infection and in broth with MIC of 10 and 1.1 μM, respectively for rifampicin (Figure 1D), and MIC of 30 and 3.3 μM, respectively for ethambutol (Figure 1F). Rifabutin and ethionamide also exhibited moderate differences of activity on Mm-inf and Mm-broth. Both rifampicin and rifabutin affected amoebae growth slightly at the highest tested concentrations, but, as illustrated for rifampicin in Figure 1C, did not prohibit Dd from reaching a plateau in its growth phase.

### Primary screening of the stilbene monomer and dimer library in a high-throughput and dual readout growth inhibition assay

**Figure 2:**
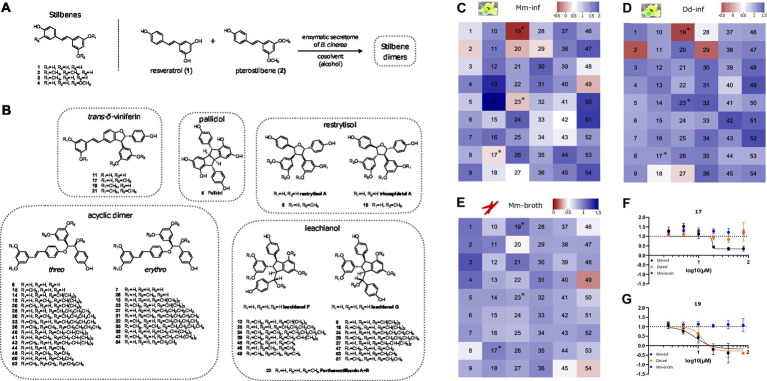
Overview of compound structures in the library and results of the screening. **(A)** Stilbene monomers (1–4) and biotransformation reaction with the enzymatic secretome of *B. cinerea*. **(B)** Structures of the stilbene dimers obtained by biotransformation of resveratrol and pterostilbene (5–54). Heatmaps in **(C–E)** color code normalized residual growth of Mm-inf **(C)**, Dd-inf **(D)** and Mm-broth **(E)** under treatment with compounds at 20 μM each. Values below the cut-off of 0.5 are displayed in shades of red, while values above the cut-off are displayed in shades of blue. Numbers in the heatmap correspond to the compounds described in panels **(A,B)**. Compounds meeting the cut-off in Mm in infection, but not in broth (i.e., Mm-inf ≤ 0.5 and Mm-broth ≥0.5) after a validation in a dose–response curve are marked with an asterisk in all three heatmaps. **(F,G)** Depict these dose–response curves of 17 and 19, respectively, of which both are analogs of the same scaffold, the *trans*-δ-viniferins. The corresponding dose–response curve for 23 can be found in Supplementary Figure S1. The log10 of the concentration in μM is shown on the *x*-axis; a dashed line at *y* = 1 represents the normalized residual growth of the vehicle control. Black points show Mm-inf, orange points Dd-inf and blue points Mm-broth. Shown are means of at least three biological replicates and the respective standard deviations. Note that in **(G)**, for visual clarity the highest dosage (80 μM, normalized residual growth of –2.2) was omitted for Mm-inf.

Next, we selected a library of compounds obtained through chemoenzymatic dimerization allowing structure activity relationship (SAR) investigations, while being relatively diverse. The compound structures can be classified into five scaffolds: *tran*s-δ-viniferin, pallidol, leachianol, restrytisol and acyclic dimer (or labruscol). Each scaffold is derived from a different coupling reaction, as described in our previous article (Huber et al., 2022a). Restrytisol, leachianol and acyclic dimer scaffolds are further classified according to their relative stereochemistry. Two parameters explain the diversity of compounds obtained in each scaffold: First, the starting materials: resveratrol, pterostilbene, or a mixture of the two, which lead to hydroxylated and *O*-methylated derivatives, and second, the cosolvent. The leachianol and acyclic dimer scaffolds are the only ones capable of incorporating a solvent molecule during their synthesis by a solvolysis reaction, i.e., a nucleophilic attack of the alcohol on an sp^2^ carbon of an intermediate. This explains the large number of members in these two series. In addition to these 50 stilbene dimers, four stilbene monomers, which were used in generating dimers or derivatives of the initial library were included in the library (resveratrol, pterostilbene, pinostilbene and isorhapontigenin, Figure 2A).

Screening directly for anti-infective activity is more stringent than screening first for antibiotic activity on Mm in broth, since the latter compounds standardly suffer from a high attrition rate due to poor pharmacokinetics. We aim at identifying anti-infective compounds, which are defined by reduced intracellular Mm growth (Mm-inf). We focus on anti-infective compounds that are ineffective on Mm in broth (Mm-broth), so-called strict anti-infective compounds, since this implies a potentially novel mode of action specific to mycobacterial infection biology. While this is the main objective, we also take into account the effect of a compound on amoeba growth (Dd-inf) and aim at identifying compounds with a high selectivity against the pathogen. For all three aspects, the hit threshold of growth reduction by at least half was applied. We subjected the library to the anti-infective and antibacterial high-throughput assays, analogously to the procedure applied in Figure 1. The hit cut-off for any readout was set at 50% of residual growth. This allowed a hit classification for each compound in the library and for each readout: Mm-inf, Dd-inf and Mm-broth. The average robust Z’-factors for this screen were 0.71 for Mm-inf and 0.74 for Mm-broth.

One of the four monomers **1–4**, pterostilbene (**2**) showed anti-infective activity (0.28 of normalized residual growth in Mm-inf), but was inactive on Mm in broth (Supplementary Figure S1B). However, this compound exhibited a strong Dd growth inhibition (–0.16 of normalized residual growth of Dd-inf), which is a possible indirect cause for its activity on Mm. Pterostilbene was tested in a DRC, revealing that activity on Dd-inf occurred already at lower concentrations than activity on Mm during infection (IC_50_ estimate of 11.1 and 19.8 μM respectively, Supplementary Figure S1B and Supplementary Table S1), weakening a possible causal link between activity on Dd and Mm during infection. The other monomers were also tested in a DRC, confirming the lack of activity of resveratrol (**1**, Supplementary Figure S1A) and isorhapontigenin (**4**, Supplementary Figure S1D) even at high concentrations, while pinostilbene (**3**, Supplementary Figure S1C) was active on Mm-inf, at high concentrations (>20 μM).

Several stilbene dimers were hits on Mm-inf, in particular **17** (with a normalized residual growth of 0.34), **19** (–0.32), **20** (0.20), **23** (0.23), **27** (0.47), **29** (0.35), **42** (0.47), and **49** (0.19) (Figure 2C and Supplementary Table S1). In broth, three of these dimers had an antibacterial activity with normalized residual growth below 0.5: **20** (0.47), **49** (0.27) and **54** (0.38) (Figure 2E), whereas **17**, **19**, **23**, **27**, **29** and **42** were shown to be anti-infectives without antibiotic activity. Among these six compounds, **19** (normalized residual growth of –0.22), **27** (0.32) and **29** (–0.10) were also inhibitors of Dd growth (Figure 2D and Supplementary Table S1). The other three dimers, **17** (normalized residual growth of 0.91), **23** (1.1) and **42** (1.2) were inactive on Dd (Figure 2D and Supplementary Table S1).

All six anti-infective dimers (**17**, **19**, **23**, **27**, **29**, and **42**) were subsequently validated in the anti-infective assay in a DRC. All of them exhibited a dose-dependent activity, but only **17**, **19**, and **23** passed again the 0.5 cut-off for Mm-inf at 20 μM (Figures 2F,G and Supplementary Figure S1E). A high anti-infective activity was confirmed for **19** (IC_50_ = 9 μM, Supplementary Table S1), but was seen as potentially correlated to a high activity on Dd growth (IC_50_ = 12.1 μM, see Supplementary Table S1). For **17**, a lower maximal effect on Mm-inf was observed (normalized residual growth of 0.3 for **17** compared to –0.2 for **19**), but also a much lower activity on Dd growth (IC_50_ = 18.1 μM for Mm-inf, an estimate for Dd-inf was not possible due to the shape of the curve, Figure 2F and Supplementary Table S1) indicating a better selectivity of **17** toward the bacteria compared to **19**. Both compounds did not show any growth inhibition of Mm-broth. Regarding compound **23**, activity at 20 μM was confirmed by a DRC with an IC_50_ of 10.4 μM and no activity on Dd growth (Supplementary Figure S1E).

Surprisingly, few compounds (**13**, **14**, **50**, **51**, and **54**) had a pro-infective activity and enhanced Mm growth during infection with a normalized residual growth value greater than 1.5 (Figure 2C and Supplementary Table S1). This feature was exclusively identified during infection and not in broth. In the scope of this study, we focused on conditions that limit Mm growth, therefore, these compounds were not further explored.

Both **17** and **19** belong to the *trans*-δ-viniferin scaffold, while **23** is an acyclic dimer. Besides **17** and **19**, which bear two *O*-methyl groups in a di-*O*-methylated ring, **11** and **21** also belong to the *trans*-δ-viniferin scaffold (Figure 2B), but with either no (**11**) or four (**21**) *O*-methyl groups. Both **11** and **21** showed no activity at 20 μM on Mm-inf with normalized residual growth of 1.1 and 1.4, respectively, or in broth with values of 1. On the acyclic dimers side, compounds **13** and **14** are both closely related to **23** but have no *O*-methyl groups and are inactive in infection (1.7, 2.0 respectively) and in broth (0.85, 0.90 respectively) (Supplementary Table S1).

In summary, the primary screen revealed **20** and **49** to be active both against extracellular and intracellular bacteria, whereas **2**, **17**, **19**, and **23** were validated anti-infectives without antibiotic properties, partly with activity against Dd (**2** and **19**), and **54** was exclusively active against Mm-broth. Pterostilbene (**2**) is a monomer, while **17** and **19** both belong to the *trans*-δ-viniferin scaffold and **23** is an acyclic dimer. Both, *trans*-δ-viniferin and the acyclic dimer families contain very similar but inactive molecules that differ in the number and position of *O*-methyl groups. Based on these different activity patterns of the *trans*-δ-viniferins we subsequently focused on exploring this set of analogs.

### High-content microscopy characterization of two hit compounds

**Figure 3:**
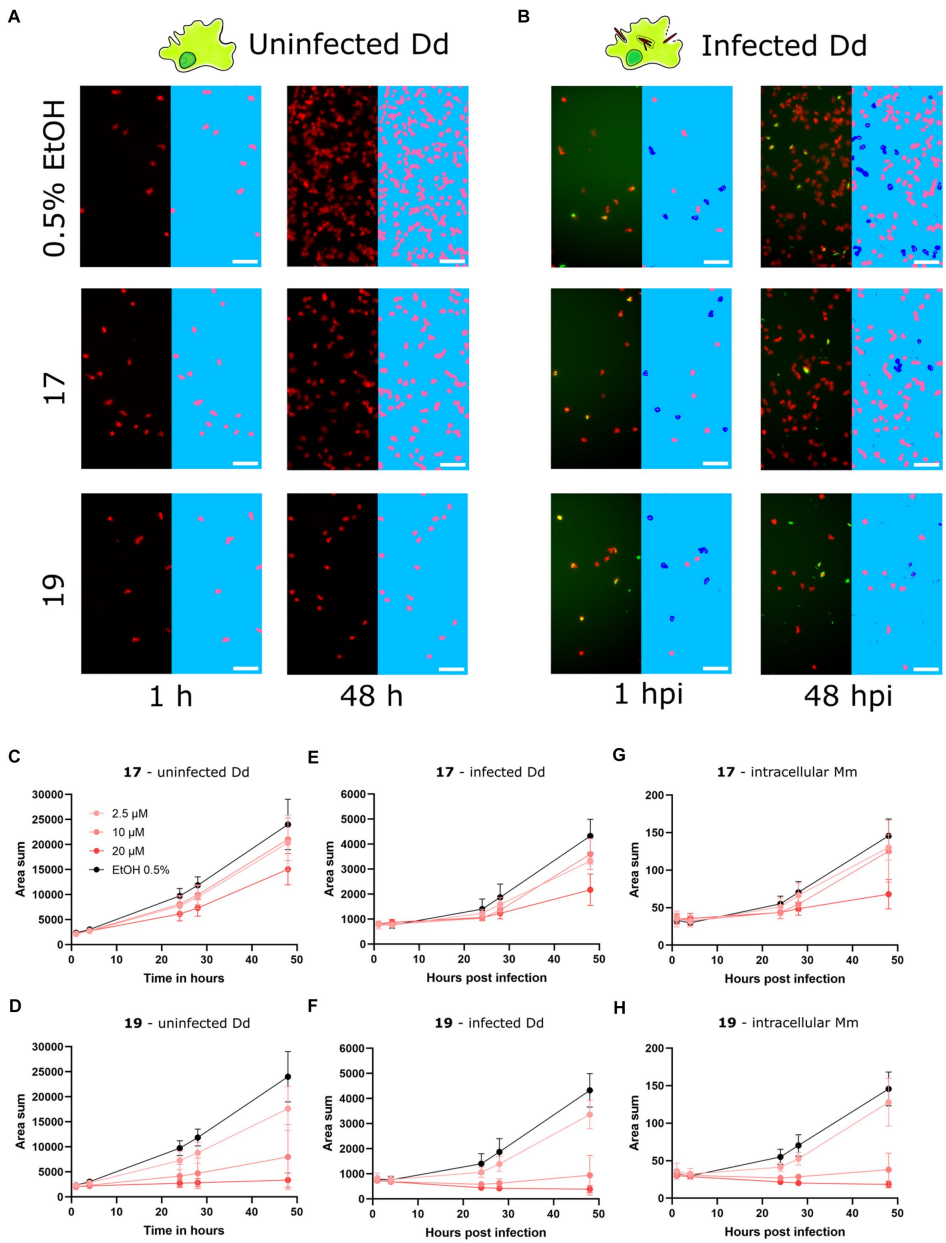
Characterization of primary hits with high-content microscopy Panels **(A,B)** show high-content microscopy panels illustrating the effects in infected and uninfected amoebae under treatment with 17 and 19 at 20 μM or 0.5% ethanol as the vehicle control at 1 and 48 h post infection (hpi). The left parts of the panels show the microscopy image, while the right parts of the panels show the corresponding segmentation. Uninfected Dd are shown in pink, infected Dd are shown in dark blue, intracellular bacteria are shown in white and extracellular bacteria are shown in black. Quantifications over the full time course and at concentrations of 2.5, 10, and 20 μM of 17 and 19 are depicted in **(C–H)**. **(C,D)** Show the segmented area of uninfected Dd over time. **(E,F)** Show the segmented area of Dd determined to be infected by Dd-Mm area overlap, over time. **(G,H)** Show the segmented area of Mm determined to be intracellular by Dd-Mm area overlap, over time. Data points represent the average of three independent biological replicates, error bars are SDs.

After identifying and validating the *trans*-δ-viniferin derivatives **17** and **19** as anti-infectives without antibiotic activity, they were further validated using time-lapse high-throughput and high-content microscopy. While the plate reader assay provided aggregated data of a partially infected cell population, High-content microscopy allowed us to focus exclusively on infected cells in the population. Both uninfected Dd and Dd infected with Mm were treated with 20, 10 or 2.5 μM of **17** or **19** and the infection was monitored by collecting microscopy images at five time points for up to 48 h. The top concentration of 20 μM was chosen to be consistent with the primary screen whereas the dilutions were chosen to observe an effect similar to the vehicle control at the lowest dose. Mm and Dd were both segmented (Figures 3A,B) and the area of Dd (Figures 3C,D), infected Dd (i.e., Dd overlapping with a bacterium, Figures 3E,F) and the area of intracellular bacteria, (i.e., Mm overlapping with Dd, Figures 3G,H) were quantified. For all quantified parameters, a dose-dependency was observed for **17** and **19**. The growth of uninfected Dd was more affected by **19** than by **17** (Figures 3C,D), while the growth of infected Dd (Figures 3E,F) and intracellular Mm (Figures 3G,H) showed the same pattern. Overall, the growth of intracellular Mm and infected Dd was almost completely inhibited by **19**, also the growth of uninfected Dd was significantly reduced. For **17**, this effect was weaker and, in addition, the growth restriction of uninfected Dd was less pronounced than the effect on intracellular Mm and infected Dd. In summary, validation of the activity of **17** and **19** using high-content microscopy confirmed that **17** was more selective for the intracellular pathogen over the host, although the overall activity was lower than that of **19**.

After validating **17** and **19** as anti-infective compounds against Mm, we wondered if an interaction between **17** and established antibiotics could be determined. We tested this compound for synergy with rifampicin and isoniazid in a checkerboard assay and additionally tested for synergy between rifampicin and isoniazid. In brief, we did not find striking synergy but rather the pro-infective effect of **17** at low dosages (see Figure 2F) dominated the antibiotic effect of both drugs (between 1.25 and 10 μM of **17**, see Supplementary Figures S3C,E).

### Evaluation of *trans*-δ-viniferin derivatives

After validating the activity of **17** and **19** by high-content microscopy, their structure–activity relationship was further investigated, as **17** and **19** showed different activity and selectivity profiles despite having very similar structures. First, the impact of their absolute configuration was investigated. Indeed, the stilbene dimers (**5–54**) are all obtained as racemic mixtures by phenoxy radical coupling. Since enantiomers often show different activities, the previously separated enantiomers of the four *trans*-δ-viniferin derivatives (Supplementary Figure S2A, **11**, **17**, **19**, **21**) (Huber et al., 2022b) were evaluated for their anti-Mm activity during infection. The results, shown in Supplementary Figures S2B–D, indicated similar activity for both enantiomers of **17** and **19**. Both enantiomers of 21 remained inactive, while a slight difference was observed between the enantiomers of **11**. The latter result has not been further investigated due to the low level of activity observed.

**Figure 4:**
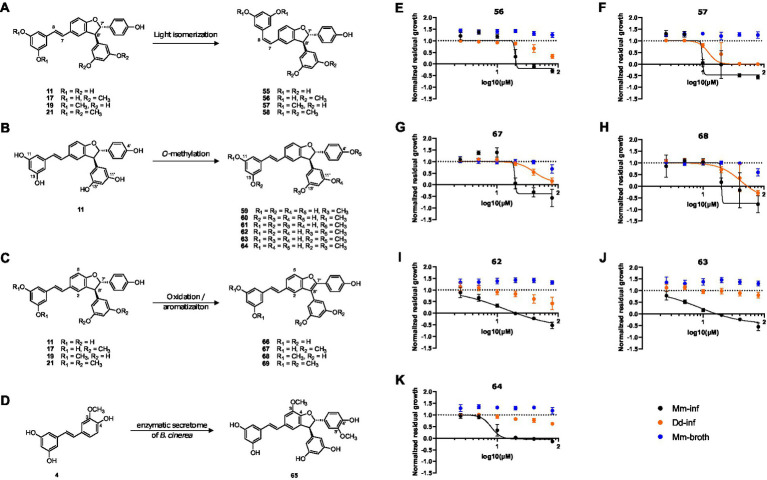
Synthesis of *trans*-δ-viniferin derivatives and corresponding structure activity relationship panel **(A)** shows light-isomerization leading to cis isomers 55–58. **(B)** Shows *O*-methylation of 11 leading to mono-*O*-methylated derivatives 59–61 and di-O-methylated derivatives 62–64. **(C)** Shows oxidation resulting in planar structures with a benzofuran moiety, 66–69. **(D)** Shows a radical coupling reaction of isorhapontigenin (4) leading to a dimer with two added methoxy groups (65) compared to *trans*-δ-viniferin (11). Panels **(E–K)** show dose–response curves of normalized residual growth of the aforementioned derivatives. **(E,F)** Show *cis* isomers 56 and 57, **(G,H)** benzofuran derivatives of 17 and 19, 67 and 68, respectively. **(I–K)** Show di-*O*-methylated derivatives 62, 63, and 64. Black points show Mm-inf, orange points Dd-inf and blue points Mm-broth; Log_10_ of the concentration in μM is shown on the *x*-axis. A dashed line at *y* = 1 represents the normalized residual growth of the vehicle control. Depicted are means of at least three biological replicates and the respective SDs.

Next, a series of derivatives of *trans*-δ-viniferin was generated and tested. These derivatives were obtained by light isomerization of the double bond (compounds **55–58**, Figure 4A), by *O*-methylation of the phenolic moieties (compounds **59–64**, Figure 4B) and by oxidation to generate benzofuran derivatives (compounds **66–69**, Figure 4C). A methoxylated *trans*-δ-viniferin derivative (**65**) was also obtained by radical coupling of isorhapontigenin (4, Figure 4D). *Cis* isomerization and oxidation were performed to change the general shape of the molecule, the former reorienting a diphenol ring, and the latter making the structure planar. On the other hand, *O*-methylation allowed to study in detail the influence of the number and position of *O*-methyl groups, whose importance has been highlighted in the primary screen by the difference in activity between compounds 11 (no *O*-methyl group, inactive), **17** and **19** (two *O*-methyl groups, active), and **21** (four *O*-methyl groups, inactive).

Among the derivatives, the two chemical reactions that change the global shape of the molecules were first investigated. In the case of a specific protein target, such a change is expected to strongly influence the activity. The *cis* isomers (Figure 4A) showed a very close activity to the parent compounds [Mm-inf IC_50_ = 18.9 μM for **17**, 19.7 μM for **56** (*cis* isomer); 9.0 μM for **19**, 9.8 μM for **57** (*cis* isomer)] (Figure 4E, F). Similarly, the *cis* isomers **55** and **58** of the inactive *trans*-δ-viniferin **11** and **21**, respectively, were also inactive at 20 μM (Supplementary Table S1). For the benzofuran derivatives, the change in activity was again weak for **17** (19.6 μM for **67**, Figure 4G and Supplementary Table S1), but a decrease in activity was observed for **19** (20.1 μM for **68**, Supplementary Table S1 and Figure 4H). The benzofuran derivative of **21** (**69**) remained inactive (Supplementary Figure S1G and Supplementary Table S1), while the corresponding derivative of **11** (**66**) showed nonspecific activity at higher dose than **17** and **19** (Mm-inf IC_50_ estimates of 29.7 μM, Dd-inf IC_50_ estimates of 26.2 μM; normalized residual growth of Mm-inf at 20 μM of 0.55) (Supplementary Figure S1F). A direct comparison with the DRC of **11** was not made because **11** was considered inactive at 20 μM in the primary screen (normalized residual growth of Mm-inf at 20 μM of 1.08). The complete IC_50_ data are given in Supplementary Table S1.

The data from the primary screen already showed that the number and position of *O*-methyl groups was important for the activity and selectivity of the compounds. Indeed, **17** and **19** which have both two *O*-methyl groups, showed different activity and selectivity in our assay. This could be due to the distinct positions of these groups. The impact of *O*-methyl groups was further supported by the decreased anti-infective activities of **11** (no *O*-methyl group) and **21** (four *O*-methyl groups). The importance of the *O*-methyl groups was therefore systematically investigated.

First, mono-*O*-methylated derivatives (**59–61**) were found to have weaker activities (less than 50% growth reduction of Mm-inf at 20 μM) and were therefore not investigated further (Supplementary Table S1).On the other hand, the di-*O*-methylated derivatives **62–64** showed an improved activity on Mm-inf (Figures 4I–K) compared to **17** and **19**. Compound **62**, showed a stronger maximal effect than **17** (–0.6 and 0.4, respectively), and its IC_50_ was slightly improved compared to **17** (13.4 and 18.9 μM, respectively, Figure 4I). Also **63** and **64** showed an improved maximal effect (−0.4 for 63 and 0 for **64**), and the IC_50_ improved by about a factor of two, compared to **17** (8.6 and 8.0 μM for **63** and **64**, respectively, Supplementary Table S1). Moreover, **62**, **63**, and **64** showed a reduced activity on the host during infection compared to **19**, while maintaining a similar effect on Mm-inf (lowest measured value of Dd-inf 0.42, 0.8 and 0.62, respectively; −0.40 for **19**). Notably, none of the modifications induced a gain of activity against Mm in broth, confirming the anti-infective property of the scaffold. On the other hand, compound **65**, corresponding to a dimethoxylated derivative of **11** (keeping the free phenol groups) did not reduce Mm growth during infection, but rather showed pro-infective activity at 20 μM and was therefore disregarded (Supplementary Figure S1).

**Figure 5:**
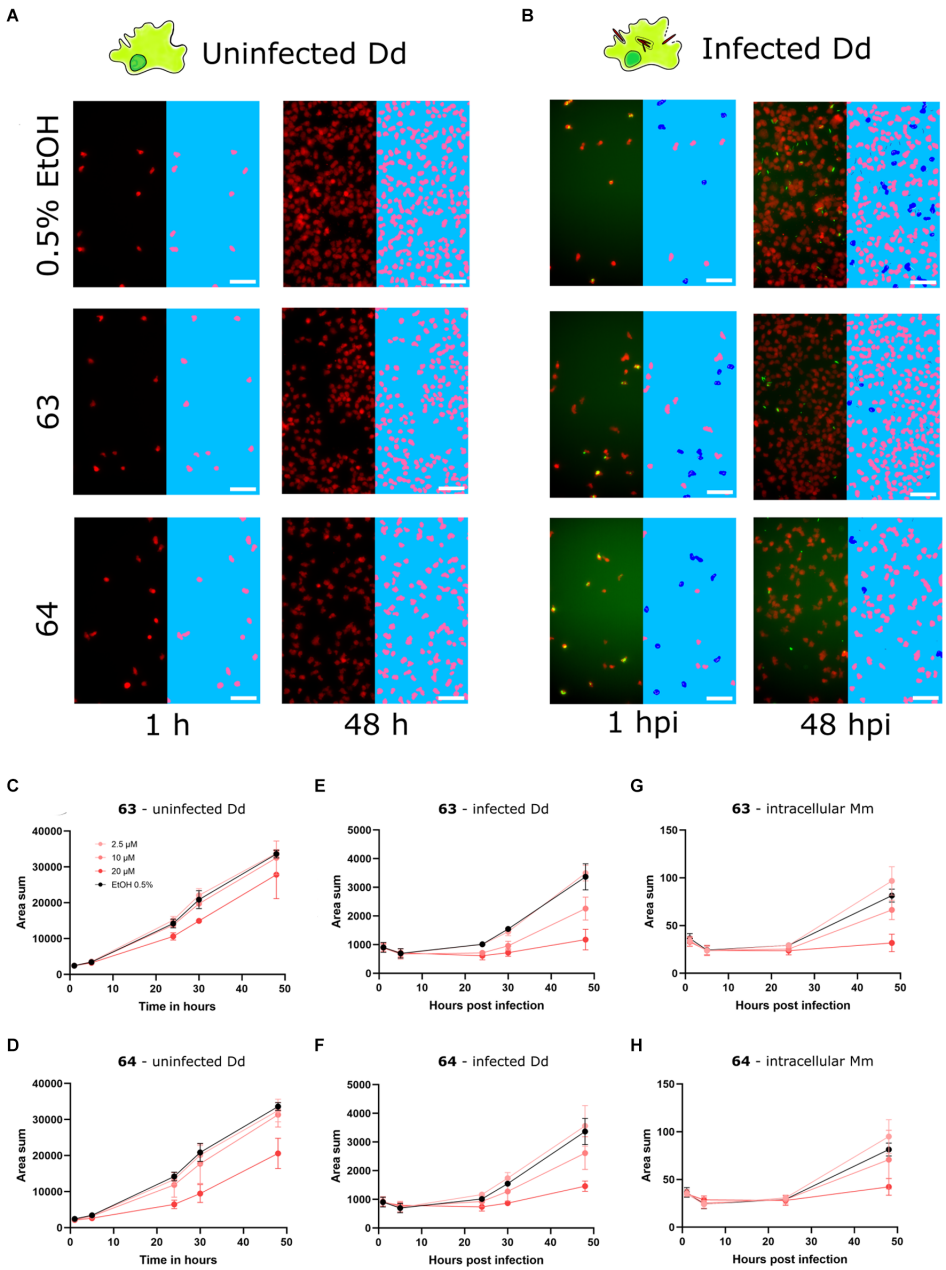
Characterization of improved derivatives with high-content microscopy Panels **(A,B)** show panels from high-content microscopy, illustrating effects in infected and uninfected amoebae under treatment with 63 and 64 at 20 μM or 0.5% ethanol as the vehicle control at 1 and 48 hpi. The left part of the panels shows the microscopy image, while the right part of the panels shows the segmented cells. Uninfected Dd are shown in pink, infected Dd are shown in dark blue, intracellular bacteria are shown in white and extracellular bacteria are shown in black. Quantifications over the full time-course at concentrations of 5, 10 and 20 μM of 63 and 64 are depicted in **(C–H)**. **(C,D)** Show the segmented area of uninfected Dd over time. **(E,F)** Show the segmented area of Dd determined to be infected by Dd-Mm area overlap, over time. **(G,H)** Show the segmented area of Mm determined to be intracellular by Dd-Mm area overlap, over time. Data points represent the average of three independent biological replicates, error bars are SDs.

After identifying **62**, **63**, and **64** as *trans*-δ-viniferin derivatives with superior potency and selectivity over the initial screening hits **17** and **19**, the activity profiles of the two most selective compounds **63** and **64** were validated using high-content microscopy (Figure 5). Similar to Figure 3, uninfected and infected Dd were treated with 20, 10, and 2.5 μM of **63** or **64** and cells were monitored for 48 h. Uninfected Dd, infected Dd and intracellular Mm were segmented (Figures 5A,B) treated with compounds and/or the vehicle. Quantification of uninfected Dd confirmed only a slight growth inhibition at 20 μM for **63** and **64** (Figures 5C,D), whereas the infection was well controlled by treatment with each of the compounds (Figures 5E–H).

Overall, the confirmation of a reduced effect of **63** and **64** on the growth of Dd compared to **19**, while having a similar potency as **19**, and higher than **17**, against Mm in infection render these two derivatives promising hits for further investigation. Interestingly, these four compounds are all *trans*-δ-viniferin isomers with two *O*-methyl groups, the only difference being their positions: both methoxy groups on a single ring for **17** and **19**, or on two different rings for **63** and **64**. The di-*O*-methylated ring connected to the conjugated double bond system in **19** seemed to contribute to activity against Dd, whereas the presence of at least one mono-*O*-methylated ring was beneficial both for maximum effect and selectivity. On the other hand, the other derivatives (*cis* isomers and benzofuran derivatives) did not allow to draw clear conclusions about the structure–activity relationship. The *cis* isomerization did not affect the activity of any of the parent compounds (**11**, **17**, **19**, and **21**), while the oxidation to benzofuran resulted in either no or only a small change (**11**, **17**, **21**) or a decrease in activity (**19**).

In addition to the aforementioned modifications, we also halogenated compounds **11**, **17**, **19**, and **21** with chlorine, bromine and iodine at different positions (Supplementary Table S1 and Supplementary Figure S4) and subsequently performed preliminary experiments of these derivatives in 96 well plates in infection (*n* = 3, *N* = 2) and in broth (*n* = 3, *N* = 1). The experiment in broth confirmed no gain of antibiotic activity of any analog (Supplementary Table S1). The experiments in infection also showed no gain of activity but revealed several inactive analogs. Although no completely consistent pattern emerged, it appeared that halogenation at the diphenol rings had the most impact on activity. In particular, double halogenation, e. g. in the case of the inactive analogs of **17**, **77** (di-brominated in positions 10 and 14) and **80** (di-chlorinated in positions 10 and 14) (Supplementary Table S1), decreases the predicted *p*ka of the hydroxyl groups from approximately 9 to almost 7, rendering them partly deprotonated in the cell. The two inactive analogs of **19**, **83**, and **89** also show at least one halogenation at the diphenol ring, whereas halogenation on the di-*O*-methylated ring, such as in **85** and **86** did not decrease activity (Supplementary Table S1).

## Discussion

In this study, we first benchmarked the high-throughput Dd-Mm infection system and subsequently deployed it in a phenotypic screen on a small library of natural product derivatives. We found *trans*-δ-viniferins to be an anti-infective scaffold, with activity specific to Mm in infection. In particular, compounds **17** and **19** stood out, which we subsequently explored by halogenation and further SAR on stereochemistry, planarity and *O*-methylation. Although we did not find striking and consistent patterns in this SAR, we confirmed anti-infective activity and higher potency for three *O*-methylated derivatives, **62**, **63**, and **64**.

During benchmarking the Dd-Mm system, we observed no activity of pyrazinamide both in infection and in broth (Figure 1J). This is in accordance with Mm being known to be naturally resistant to pyrazinamide (Aubry et al., 2000; Finkelstein and Oren, 2011). Resistance to isoniazid has also been reported (Aubry et al., 2000; Lorian, 2005), and in both assays isoniazid had the highest observed MIC (30 μM or 4.1 μg/mL). The National Committee for Clinical Laboratory Standards (NCCLS) defined breakpoints of antibacterial susceptibility for MIC_90_ from clinical isolates. Breakpoints for Mm were defined for rifabutin (2 μg/mL), ethambutol (4 μg/mL) (Woods and Clinical and Laboratory Standards Institute, 2011) and ethionamide (5 μg/mL) (Lorian, 2005). MICs observed in this study are all below the respective breakpoints, with the exception of ethambutol in infection (30 μM or 6.1 μg/mL). Indeed, ethambutol and rifampicin showed a large difference of activity between infection and growth in broth. Such a pattern was reported for rifampicin in Raw264.7 (Brodin et al., 2010) and THP-1 macrophages, due to export from the host cell via P-gp, but specifically not for ethambutol (Hartkoorn et al., 2007). Dd possesses an array of ABC transporters and is known for rapid drug export (Anjard et al., 2002; Miranda et al., 2013), emphasizing its stringency as a drug screening model in regard to pharmacokinetics.

After screening a library of stilbene dimers in the Dd-Mm system, we detected mostly anti-infective activities in contrast to classical antibiotic compounds, characterized by no activity in broth throughout the tested concentration range. Subsequently, we investigated the scaffold via targeted SAR and found derivatives with improved selectivity and maximal effect. This demonstrates not only the power of the Dd-Mm system as a 3R infection model, but also the value of exploring natural chemistry by novel approaches of derivatization. The observed anti-infective phenotype might be caused by accumulation inside Dd, which we speculate is unlikely, since Dd is known to rapidly export xenobiotics through its array of ABC transporters (Anjard et al., 2002; Miranda et al., 2013), as it seems to be the case for rifampicin. An anti-infective phenotype is thus likely to be caused by an antivirulence or host-directed defence booster mode of action (Gries, 2024).

Previous work has identified host-directed compounds in mammalian cell systems, and some have advanced to clinical studies (Huang et al., 2024). Several studies have shed light on the potential of tyrosine kinase inhibitors for TB treatment. For example, therapeutic doses of imatinib have proven effective in enhancing phagosome acidification and maturation, leading to a reduction in intracellular Mtb survival, both *in vitro* and in infected mice (Napier et al., 2011; Bruns et al., 2012). Another HDT approach involves metformin, a medication commonly prescribed for type 2 diabetes, which activates AMP-activated protein kinase. Metformin not only reduces the bacterial burden but also improves lung pathology in both mice and humans by enhancing autophagy and increasing the production of reactive oxygen species (Singhal et al., 2014). An emerging avenue focuses on inhibiting Mtb’s intracellular acquisition of fatty acids. Tetrahydrolipstatin (THL), which is recognized as an inhibitor of pancreatic lipase and marketed as a weight loss aid, shows promise as an anti-Mtb compound. Although the precise mechanism of its action is only partially understood, it is believed that THL depletes essential lipids from the intracellular environment, which are critical for Mtb’s survival (Parker et al., 2009).

Members of the group of compounds with a trans-δ-viniferin scaffold (11, 17, 19, 21.) exerted the strongest anti-infective effect in our assay. Members of this family are phytoalexins found in various plants, including grapevines (*Vitis vinifera* L.), and they act as defense compounds produced by plants in response to stress, infection, or other environmental challenges, including microbial pathogens (Langcake and Pryce, 1976). Phenolic compounds such as stilbenes and the *trans*-δ-viniferins presented here have many reported biological activities against bacteria (Langcake and Pryce, 1976; Vestergaard and Ingmer, 2019; Righi et al., 2020), but also diverse positive effects on mammalian cells (Rimando et al., 2005; Qureshi et al., 2012; Li et al., 2016; Ren et al., 2018).

In a recent study, investigating essential requirements for infection of Mm in Dd via Transposon sequencing (Tn-Seq), we highlighted genes and pathways that might act as targets for antivirulence compounds (Lefrançois et al., 2024). Members of lipid metabolism are prominent candidates, such as genes required for phthiocerol dimycocerosates (PDIMs) synthesis (Quigley et al., 2017) or the *Mce* operons, which are associated with lipid transport (Klepp et al., 2022).

The host membrane might also be altered in a way that makes it less sensitive to the bacteria membrane damaging machinery, for example by interfering with cholesterol in the MCV membrane. Cholesterol has been discussed to play a role in membrane damage, bacterial entry and also during infection of other intracellular, vacuolar pathogens (Gatfield and Pieters, 2000; Howe and Heinzen, 2006; De Jonge et al., 2007). In addition, in Dd a knock-out of vacuolins, the functional homologues of the mammalian flotillins which organize membrane microdomains rich in sterols, lead to intracellular growth attenuation of Mm (Bosmani et al., 2021).

Since we did not observe a striking shift in IC_50_ upon manipulating the shape of the *trans*-δ-viniferins **17** and **19** via planarisation (benzofuran derivatives) and *cis*-isomerisation of the double bond, we speculate that the mechanism of action is unlikely to follow the conventional pattern of selective binding to a catalytic pocket on a protein but may indeed rely on interference with cellular membranes, as discussed above.

However, *trans*-δ-viniferins have been reported as Wnt modulators, which in turn is hypothesized to be a target for pharmaceutical autophagy induction (Huber et al., 2022a). Interestingly, autophagy is also discussed as a central process of cell autonomous defence against Mtb and Mm (Aylan et al., 2023; Golovkine et al., 2023).

Since *trans*-δ-viniferins show activities in many biological systems, this class of compounds might be described as pan assay interference compounds (PAINs) (Baell, 2016). PAINs are compounds that in principle can be developed into drugs, but due to their promiscuous target interactions in a less systematic manner and thus at a higher cost. For example, catecholamine drugs contain catechols, which are considered a classical feature of a PAIN (Baell, 2016). Other structural features of PAINs, such as a flexible three-dimensional shape and reactive side groups are present in *trans*-δ-viniferins. Nevertheless, we observed striking specific activity on intracellular Mm for **17**, **62**, **63**, and **64**. In addition, current developments in pharmaceutical research try to embrace drugs with multiple targets, facilitated by advancing techniques to decipher complicated mechanisms of action, possibly allowing to progress putative PAINs more systematically, in the future.

A few structures were also found to enhance Mm growth during infection, but not in broth. During infection, 13, 14, 50, 51, 54 resulted in a normalized residual growth value greater than 1.5 (Figure 1C and Supplementary Table S1). Compounds that promote growth during infection, so-called pro-infectives may also be of fundamental interest. Although these compounds are generally ignored in most screenings and have limited value in drug discovery, they could be potential discovery tools to understand the mechanisms restricting intracellular mycobacterial survival (Subhash and Sundaramurthy, 2021). In fact, such compounds might act by disabling cell-autonomous defences and thus might enhance our understanding of innate immune mechanisms. As our main objective in this study is to focus on compounds that limit Mm growth, we did not further explore pro-infective compounds.

In summary, we emphasize the value of using nature inspired methods to derivatize simple scaffolds to bioactive compounds. Additionally, we demonstrated the effectiveness and potential of the Dd-Mm infection system for drug discovery, a platform fully compliant with the 3R model. This could be considered an added value to the drug discovery pipeline, offering an affordable and ethically sound system that complements the primary efforts in Mtb drug discovery. A remaining question is the validation of hit compounds in the Mm-Zebrafish model to confirm their efficacy in a more complex vertebrate model system.

## Data Availability

The original contributions presented in the study are included in the article/Supplementary material, further inquiries can be directed to the corresponding authors.
